# Mass Spectrometry-Based Proteomic and Metabolomic Profiling of Serum Samples for Discovery and Validation of Tuberculosis Diagnostic Biomarker Signature

**DOI:** 10.3390/ijms232213733

**Published:** 2022-11-08

**Authors:** Ana Filipa Fernandes, Luís Gafeira Gonçalves, Maria Bento, Sandra I. Anjo, Bruno Manadas, Clara Barroso, Miguel Villar, Rita Macedo, Maria João Simões, Ana Varela Coelho

**Affiliations:** 1 Instituto de Tecnologia Química e Biológica António Xavier, Universidade Nova de Lisboa, 2780-157 Oeiras, Portugal; 2 CNC-Center for Neuroscience and Cell Biology, University of Coimbra, 3004-504 Coimbra, Portugal; 3 CIBB—Centre for Innovative Biomedicine and Biotechnology, University of Coimbra, 3004-504 Coimbra, Portugal; 4 III Institute for Interdisciplinary Research, University of Coimbra (IIIUC), 3004-504 Coimbra, Portugal; 5 CDP Almada-Seixal, ARSLVT, 2805-021 Almada, Portugal; 6 CDP Venda Nova, ARSLVT, 2700-220 Amadora, Portugal; 7 Instituto Nacional de Saúde Dr. Ricardo Jorge, 1649-016 Lisboa, Portugal

**Keywords:** tuberculosis, diagnosis, biomarkers, multiomics, mass spectrometry, blood serum

## Abstract

Tuberculosis (TB) is a transmissible disease listed as one of the 10 leading causes of death worldwide (10 million infected in 2019). A swift and precise diagnosis is essential to forestall its transmission, for which the discovery of effective diagnostic biomarkers is crucial. In this study, we aimed to discover molecular biomarkers for the early diagnosis of tuberculosis. Two independent cohorts comprising 29 and 34 subjects were assayed by proteomics, and 49 were included for metabolomic analysis. All subjects were arranged into three experimental groups—healthy controls (controls), latent TB infection (LTBI), and TB patients. LC-MS/MS blood serum protein and metabolite levels were submitted to univariate, multivariate, and ROC analysis. From the 149 proteins quantified in the discovery set, 25 were found to be differentially abundant between controls and TB patients. The AUC, specificity, and sensitivity, determined by ROC statistical analysis of the model composed of four of these proteins considering both proteomic sets, were 0.96, 93%, and 91%, respectively. The five metabolites (9-methyluric acid, indole-3-lactic acid, trans-3-indoleacrylic acid, hexanoylglycine, and N-acetyl-L-leucine) that better discriminate the control and TB patient groups (VIP > 1.75) from a total of 92 metabolites quantified in both ionization modes were submitted to ROC analysis. An AUC = 1 was determined, with all samples being correctly assigned to the respective experimental group. An integrated ROC analysis enrolling one protein and four metabolites was also performed for the common control and TB patients in the proteomic and metabolomic groups. This combined signature correctly assigned the 12 controls and 12 patients used only for prediction (AUC = 1, specificity = 100%, and sensitivity = 100%). This multiomics approach revealed a biomarker signature for tuberculosis diagnosis that could be potentially used for developing a point-of-care diagnosis clinical test.

## 1. Introduction

Tuberculosis (TB), caused by *Mycobacterium tuberculosis* (Mtb), is still a global pandemic despite being completely curable. According to the WHO, in 2020, 10 million new TB cases were reported and there were 1.5 million deaths, making it the second leading infectious killer after COVID-19 [1,2]. Failure of effective vaccine protection, lack of early detection of the disease, emergence of drug resistance, deadly synergism with HIV infection, increasing global international migration flows, and the SARS-CoV-2 pandemic have limited the success of TB disease management. In order to overcome these frightful situations, the “End TB Strategy” defined as a priority the “early diagnosis of TB, and systematic screening of contacts and high-risk groups” [1]. In the last years, TB diagnosis improved with the development of new rapid diagnostics based on nucleic acid amplification tests (NAATs) and online-probe assays (LPA), which are DNA-strip-based tests. However, these new diagnostic tests require specific equipment and knowledge not broadly available in endemic settings [3,4]. In adults, with a prior history of TB and treatment completion within the last 5 years or for repeated testing, there is low certainty of evidence for NAAT test accuracy associated with a high false-positivity rate [4]. Additionally, LPA tests are not recommended for the direct testing of sputum smear-negative specimens. As such, TB diagnosis is still dependent on the microbiological detection of Mtb from clinical samples, which requires physical biosafety laboratories and demanding and time-laborious procedures, and lacks sensitivity in paucibacillary cases, leading to late TB diagnosis [5]. Clinical diagnosis based on symptoms, abnormalities on chest radiography, or suggestive histology is still important for TB diagnosis. Extrapulmonary TB (EPTB) diagnosis is even more challenging than pulmonary TB (PTB) since it requires more invasive diagnostic sampling with more risks and costs [6]. On the side of the host, several biomolecules detected in TB patients (specific transcriptional signature, micro-RNAs, antibodies, and interferon inducible proteins) were tested, but the simultaneous measurement of a large set of genes, variable accuracy, and the heterogeneity to antibody response proved to be suboptimal [3,5]. Additionally, monitoring of TB treatment is still on the sputum-based culture that, as already mentioned, has its drawbacks in terms of time-consumption, especially in these cases with very low Mtb loads in the sputum samples [7]. There has been a call for the development of new quantitative, non-sputum, nonculture-based TB biomarkers suitable for monitoring TB therapeutics. Lately, the urgent need for new non-DNA biomarkers has also been consistently referenced [3]. In addition, most likely, diagnostic specificity performance will be improved using multi-biomarker signatures instead of a single biomarker. The authors of [7,8,9] suggested that “combining resources and evaluating multiple biomarkers side-by-side” could be a way to achieve this goal. Untargeted approaches are the more suitable strategy for a broad discovery and evaluation of meaningful biomarkers. Among these are proteomics and metabolomics, which allow the collective quantification of pools of biomolecules, expanding the universe of compounds under study and also helping solve one of the major difficulties in establishing an accurate TB diagnosis test, the partial understanding of the complex host–pathogen interaction [10]. Relevant findings for TB diagnosis using these strategies are summarized in recent reviews; however, their value is diminished by cohort and methodological variabilities [8,11,12]. Moreover, although publications of new biomarkers are common, follow-up studies on the refinement, validation, and independent confirmation of them are not. In a previous study from our team, a relevant biomarker signature for active TB, extending and integrating previously published results, was discovered in blood serum by NMR metabolomics [13]. Our results indicated that there are a common set of TB biomarkers in pulmonary TB and EPTB patients’ serum. A set of these metabolites (hypoxanthine, asparagine, methionine, citrate, mannose, 3-hydroxyisobutyrate, glutamate, and lysine) were used to successfully predict serum samples from an Indian cohort [13]. In the present study, an alternative biomarker signature composed of four metabolites and one protein was defined by LC-MS/MS based proteomics and metabolomics.

## 2. Results

**Figure 1:**
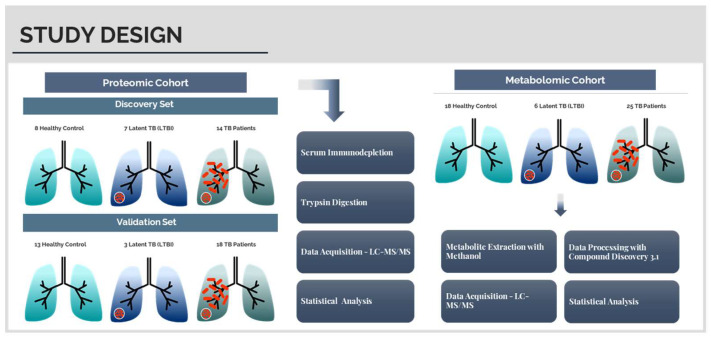
Design of the study, including a proteomic and a metabolomic cohort and the experimental workflow.

A combined approach of LC-MS-based proteomics and metabolomics was employed in this study to find a new signature of tuberculosis biomarkers in serum. For that, two different cohorts were used: one to assess new serum proteins that can be used as TB biomarkers (discovery set) and the other to validate the results obtained (validation set). For the metabolomic study, the two cohorts were merged. The cohorts were divided into three experimental groups: 1. Healthy individuals, with a negative IGRA test (control group); 2. Healthy, latently infected individuals who tested positive to the IGRA test (LTBI group); and 3. Patients diagnosed with pulmonary and extrapulmonary tuberculosis (TB group) (see Figure 1).

### 2.1. The Protein Profile of Tuberculosis Patients Is Distinct from That of Healthy Controls

The protein profile of each set was analyzed using LC-MS/MS and allowed the quantification of 149 proteins (with ≥2 peptides) in the discovery set (Supplementary Table S1) and 79 in the validation set (with ≥2 peptides) (Supplementary Table S2). All proteins quantified in the validation set were also present in the discovery set, with the exception of the immunoglobulin-heavy constant mu (P01871) that was only quantified in the validation set.

**Figure 2:**
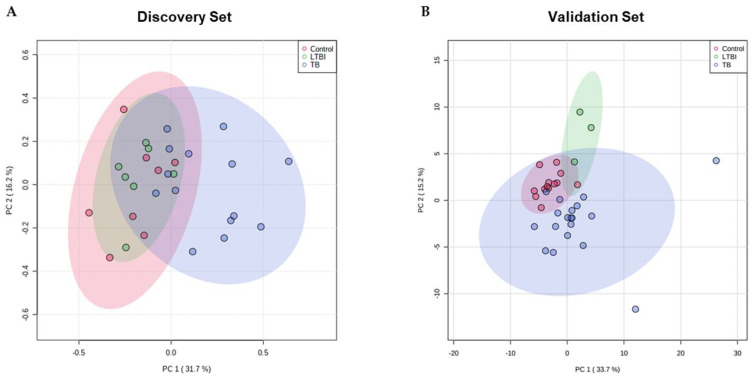
Serum proteomic PCA for the discovery and validation sets. PCA scores plot for the first and second components for the three experimental groups: control (red), LTBI (green), and TB (blue) for the discovery (**A**) and validation (**B**) sets. Areas in red, green, and blue represent the 95% confidence region. Dots within each area represent the replicates for each condition: n(control) = 8, n(LTBI) = 7, and n(TB) = 14 in the discovery set, and n(control) = 13, n(LTBI) = 3, and n(TB) = 18 in the validation set.

**Figure 3:**
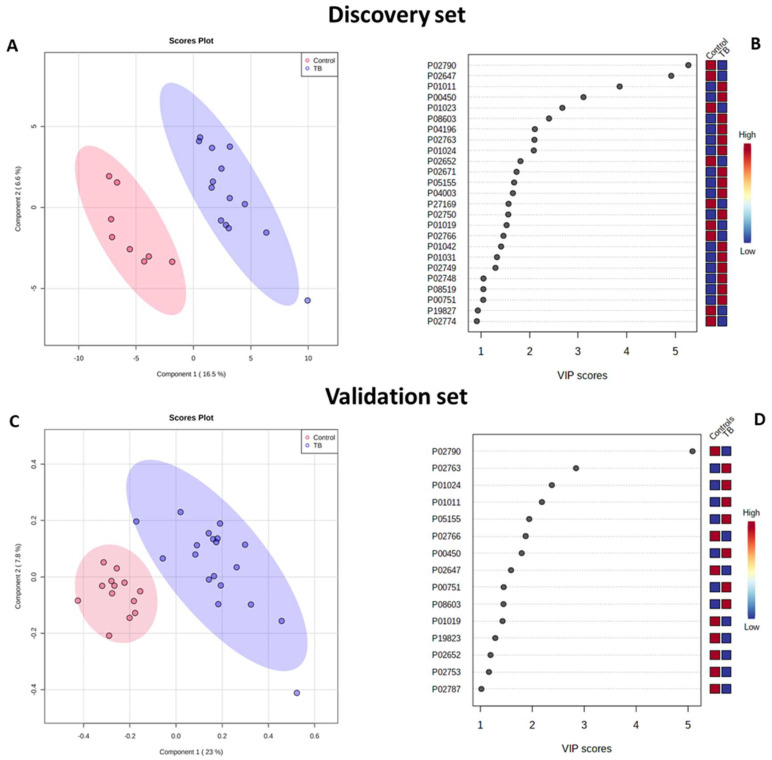
Serum proteomics PLS-DA model discriminates between controls and TB patients in the (**A**,**B**) discovery and (**C**,**D**) validation sets. (**A**,**C**) PLS-DA scores plot for the first and second components constructed with controls and TB patients serum proteins (for the discovery set: Q^2^ = 0.841, permutations (2000) *p* = 0.018, n(control) = 8 and n(TB) = 14; for the validation set: Q^2^ = 0.754, permutations (2000) *p* = 0.005, n(control) = 13 and n(TB = 18). Areas in red and blue represent the 95% confidence region. Dots represent the replicates for each condition. (**B**,**D**) Proteins responsible for the discrimination in the first component (VIP > 1.0).

With regard to the discovery set, it is possible to observe that in the PCA scores plot, TB patient samples discriminate better from the samples from the other two groups (control and LTBI) than those from each other (Figure 2). Distinct serum protein contents were not determined between controls and LTBI. A PLS-DA model using the three experimental groups showed low discriminatory power (Q^2^ = 0.446) (see Supplementary Figure S1). PLS-DA analysis showed discrimination between controls and TB patients (Q^2^ = 0.841; permutations (2000) *p* = 0.018) (see Figure 3), but not between LTBI and TB patients (Q^2^ = 0.361) (see Supplementary Figure S2A). The protein analysis of control vs. LTBI also revealed a nonvalid PLS-DA model between the groups (see Supplementary Figure S2B). In the projection of PLS-DA models, 25 proteins contributed significantly (VIPs > 1) for the discrimination of controls and TB patients. Of those, nine proteins were decreased and sixteen were increased in the TB patients group (Table 1).

Similar results were obtained for the multivariate analysis performed for the control and TB patients groups of the validation and discovery sets (see Figure 3). The 12 discriminant proteins with VIP > 1.5 are the same in both sets. These results demonstrate that both cohorts are comparable.

In the volcano plot for the protein serum levels of the discovery set, only three proteins met the defined criteria (FC > ±2 and corrected *p* < 0.05). Two were increased in TB patients’ serum: complement factor H-related protein 5 (Q9BXR6) and C4b-binding protein alpha chain (P04003), and the serotransferrin levels were decreased (P02787). These proteins belong to the same pathways found relevant for the discriminant proteins identified by multivariate analysis. Its potential involvement in TB pathophysiology is discussed below.

#### Development of a Proteomic Diagnostic Model

ROC analysis was performed to assess the potential of each protein as a TB diagnostic biomarker. In this analysis, the discovery set was used to construct the ROC curve; the validation set samples were split and used as hold-out and as predictors.

**Figure 4:**
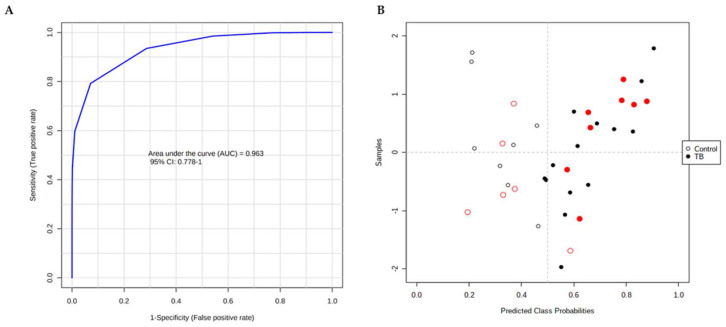
ROC constructed for the proteomic discovery set accurately assigns the validation set samples. (**A**) The combination of four proteins that presented an AUC above 0.92 (P01011, P00450, P08603, and P02790) were used to construct the ROC using a PLS-DA model with 3 latent variables. (**B**) Sample distribution within the model. Samples used for the model building in black and the hold-out samples in red. Open and filled circles represent control and patient samples, respectively.

For the ROC curve, four proteins were selected that presented an AUC above 0.92: alpha-1-antichymotrypsin (P01011), ceruloplasmin (P00450), hemopexin (P02790), and complement factor H (P08603). The model created by these four proteins displayed a high discriminative capacity with respect to the control group and the patient group, with an AUC = 0.963 with a specificity of 93% (with only 1/14 of controls being wrongly identified as patients) and a sensitivity of 91% (with 2/22 of patients identified as controls) (see Figure 4). The model generated correctly identified all the TB samples and only misclassified a control sample in the validation set.

### 2.2. Comprehending the Metabolic Behavior of Tuberculosis Patients and Healthy Controls

**Figure 5:**
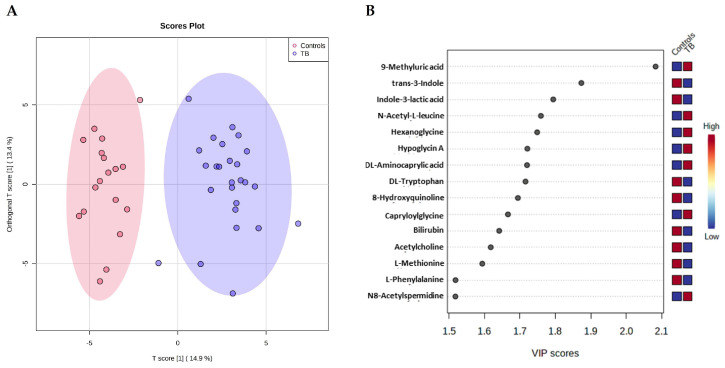
OPLS-DA model based on the metabolites quantified by LC-MS discriminates TB patients from the control group. (**A**) Score plot of the OPLS-DA model shows a clear discrimination between the serum metabolites levels of patient and control groups (Q^2^ = 0.733, permutations (2000) *p* < 0.001). Areas in red and blue represent the 95% confidence region. Dots represent the replicates for each condition (n(control) = 18 and n(TB) = 25). (**B**) The metabolites that are important for the discrimination between the two groups (VIP > 1.5 in the first component).

Using Compound Discoverer™ 3.1 software (ThermoFisher Scientific), 69 metabolites were detected in positive mode (Supplementary Table S3) and 32 metabolites in negative mode (Supplementary Table S4), as well as the internal standard—3-nitro-L-tyrosine—in both modes. There are nine compounds that were detected in both modes (4-acetamidobutanoic acid, DL-arginine, L-histidine, N-phenylacetylglutamine, L-tyrosine, DL-tryptophan, 4-guanidinobutyric acid, α-aspartylphenylalanine, and L-phenylalanine). PCA analysis does not discriminate among the three experimental groups (Supplementary Figure S3). The OPLS-DA model created using all the metabolites (a total of 92 metabolites) only discriminates the patients with active TB from the control group (Figure 5A) (Q^2^ = 0.733, permutations (2000) *p* = 0.001), as previously also reported by the analysis of our proteomic data. The most important metabolites for TB discrimination (VIP > 1.5) are 9-methyluric acid, N-acetyl-L-leucine, and hexanoglycine that are increased in TB patients, and trans-3-indoleacrylic acid and indole-3-lactic acid that are decreased in TB patients (Figure 5B).

**Figure 6:**
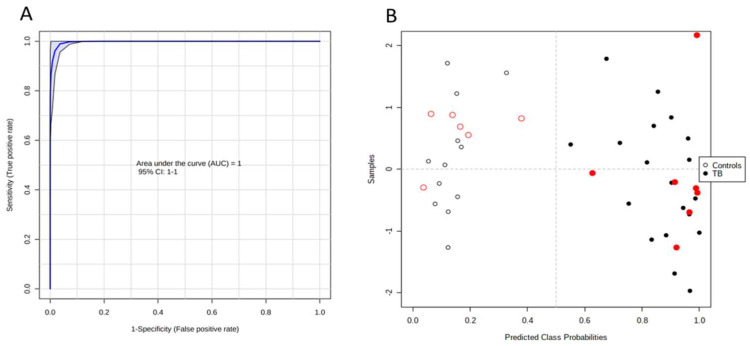
ROC analysis based on the metabolomic data. (**A**) The combination of five metabolites (9-methyluric acid, indole-3-lactic acid, trans-3-indoleacrylic acid, hexanoylglycine, and N-acetyl-L-leucine) present an AUC = 1 using a linear SVM model. (**B**) Sample distribution within the model; samples used for the model are in black and the hold-out samples are in red. Open and filled circles represent control and TB samples, respectively.

All the metabolites quantified by LC-MS/MS analysis were used to perform an ROC analysis. Five of the metabolites with an AUC > 0.9 (9-methyluric acid, indole-3-lactic acid, trans-3-indoleacrylic acid, hexanoylglycine, and N-acetyl-L-leucine) display a high discriminating capacity with an AUC = 1, a specificity of 100%, and a sensitivity of 100% (see Figure 6). In addition, the class was accurately attributed to the six patient and six control samples that were randomly selected to be used as hold-out.

### 2.3. Biomarkers for Tuberculosis Combining the Proteomic and the Metabolomic Data

**Figure 7:**
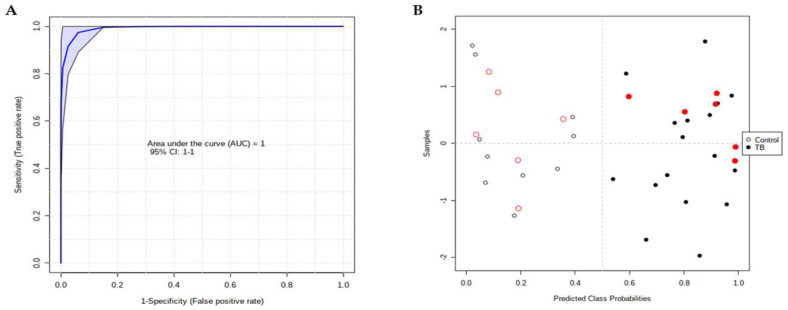
ROC analysis based on proteomic and metabolomic data. (**A**) The combination of four metabolites (indole-3-lactic acid, trans-3-indoleacrylic acid, hexanoylglycine, and N-acetyl-L-leucine) and one protein (hemopexin) present an AUC = 1 using a linear SVM model. (**B**) Sample distribution within the model; samples used for the model are in black and the hold-out samples are in red. Open and filled circles represent control and patient samples, respectively.

The several cohorts used in this study were combined to test a new set of biomarkers based on the proteomic and metabolomic data. In common between the samples used for proteomic and metabolomic analysis, there are 16 control samples and 23 patient samples that were used for the ROC analysis. The previous four proteins of ROC analysis (Figure 4) and the five metabolites with a VIP > 1.75 in OPLS-DA analysis (Figure 5) were used as variables. From that set of biomarkers, four metabolites—indole-3-lactic acid, trans-3-indoleacrylic acid, hexanoylglycine, and N-acetyl-L-leucine—and one protein, hemopexin, presented an AUC above 0.9 and were used in further analysis. The ROC obtained using a linear SVM model presented an AUC = 1 and accurately attributed the class to the six patient and six control samples that were randomly selected to be used as hold-out (Figure 7). Moreover, for this combination of biomarkers, the calculated false discovery rate obtained with MetSizeR package for our sample size is lower than 0.05.

## 3. Discussion

PLS-DA analysis at the serum proteomic level for the discovery and validation sets and at the metabolomic level showed discrimination between TB patients and the control group. No discrimination between the LTBI group and the other groups was found. These observations are common to several other studies [13,14,15].

### 3.1. Complement System and Cholesterol Metabolism Are Incremented in Active Tuberculosis

In the discovery set by multivariate analysis, 25 proteins were found to discriminate between control and patient groups, and 16 of those were increased in patients. Twenty of these proteins were found in blood microparticles’ composition according to Gene Ontology. Based on Kegg pathways analysis, ten proteins are involved in the complement and coagulation cascades and five in cholesterol metabolism. Higher abundance was determined for proteins belonging to these two pathways in TB patients after rifampin/rifapentine, isoniazid, pyrazinamide, and ethambutol treatment [7].

The complement system is activated during a challenge with *M. tuberculosis*; however, its exact involvement it is not understood yet [14,16], although a lower abundance was also reported for proteins associated with complement activation in active TB compared to healthy controls, together with the contradictory increment of immunoglobulins responsible for the initial triggering of complement [17]. In the same study, complement activation was reduced in 2 months’ intensively treated TB cases compared with untreated TB cases. This finding is in accordance with our results. From this pathway, all detected proteins in our study are incremented in TB patients, except alpha-2-macroglobulin. Six proteins (plasma protease C1 inhibitor, C4b-binding protein, complement factors B and H, and complement components 3, 5, and 9) are involved in the complex immune surveillance system responsible for the defense against microbes, labeling them for phagocytosis [18]. Complement factor H-related protein 5 was also found to be incremented in TB by volcano plot analysis. In a recent study, it was reported that the levels of the complement component C1q and of plasma protease C1 inhibitor (C1-INH) were increased in TB patients’ serum when compared with healthy controls [19,20,21]. Lubbers et al. (2020) found a positive correlation between the levels of both proteins and high specificity for active TB, although a low sensitivity was reported. C1-INH levels dropped after two and six months of TB treatment [19]. It was also determined that a significant increase in C1-INH levels is not related to other infections or inflammation [19]. By immunoassay, complement factor H was defined as the best single biomarker for active TB [22]. A proteomic study aiming to discover biomarkers for osteoarticular tuberculosis by comparison with other rheumatic diseases showed that complement factor H-related proteins 1 and 2 were only upregulated in the tuberculosis group [23].

During infection, Mtb survives in hypoxia, and several metabolic processes suffer adjustments, namely, the cholesterol metabolism, since Mtb shifts to host-derived cholesterol and lipids as primary nutrient sources [24]. Low-density lipoproteins are sequestered within macrophages; their cholesterol accumulates as droplets, leading to the formation of TB characteristic foamy macrophages, which may be essential for Mtb persistence [25]. Related with cholesterol metabolism, two proteins were found to be increased in TB patients (beta-2-glycoprotein 1 (APOH) and apolipoprotein(a) (LPA)), while three were more abundant in controls (apolipoprotein A-I (APOA1), apolipoprotein A-II (APOA2) and serum paraoxonase/arylesterase 1 (PON1)). The latter are involved in the handling of serum-circulating cholesterol storage lipoproteins. APOA1/2 were already reported to be decreased in TB patients [14].

### 3.2. Iron Metabolism Is Dysregulated in Active TB

The assimilation of iron by *M. tuberculosis* has been suggested as a key pathway for the progression of TB [26]. The mycobacterium survival implies competition for iron uptake with the host. In the present study, three proteins associated with iron metabolism were found to be differentially abundant between patient and control. Serotransferrin and hemopexin are decreased, and ceruloplasmin is incremented in patient blood serum. As in the present study, serotransferrin and hemopexin levels have previously been found to be decreased in TB patients [7,14,27]. After intestinal absorption, ferric iron (Fe^3+^) is transported by serotransferrin to storage and utilization sites. Iron enters the cell by endocytosis and it is reduced to ferrous iron (Fe^2+^). In the mitochondria, iron is a fundamental component of heme and iron–sulfur-cluster-containing proteins, which have central roles in the electron transport chain [25]. Ceruloplasmin promotes the reoxidation of iron when it re-enters blood circulation. To reduce toxicity, due to heme tissue accumulation, hemopexin binds and transports it to the liver for catabolism and excretion. A possible explanation for the reduction of serotransferrin levels is the competition with the Mtb iron scavenging system, which will induce a reposition of iron levels in circulation and, consequently, an increment of ceruloplasmin activity. Serum transferrin and hemopexin often decline together during acute-phase reactions and malnutrition [28].

### 3.3. Blood Serum Metabolome Changes on Active Tuberculosis

The OPLS-DA generated with the levels of the metabolites in serum determined by LC-MS of controls vs. patients indicated that 9-methyluric acid, N-acetyl-L-leucine, and hexanoylglycine (increased on TB patients), and trans-3-indoleacrylic acid and indole-3-lactic acid (decreased in TB patients) are the most important metabolites for the discrimination. Two of them are related to the tryptophan metabolism by gut bacteria: trans-3-indoleacrylic acid and indole-3-lactic acid [29]. Indole-3-lactic is produced by *Bifidobacterium* species in the human gut and it is described as having an anti-inflammatory effect [30]. The same type of property was described for trans-3-indoleacrylic acid, a compound produced by *Peptostreptococcus* species [31]. Also relevant for the discrimination between patient and control (VIP > 1.5) are the decreased levels of L-tryptophan and L-phenylalanine (Figure 5B), another aromatic amino acid. L-tryptophan blood levels were also reported to decrease in previous studies [32,33], while the opposite variation was described for phenylalanine [32]. L-tryptophan was increased after 6 months of TB treatment [34]. This amino acid, together with L-arginine and L-glutamine, has been stated as a key regulator of immunometabolism in TB [35]. Its decreased levels in TB is probably related to the fact that tryptophan is an amino acid essential to *M. tuberculosis* growth and is in accordance with the increment of L-tryptophan catabolism in response to Mtb infection [25]. Interestingly, Luier et al. (2016) reported higher levels of tryptophan catabolites in the urine of TB patients relative to healthy controls [36].

The other three differential metabolites (9-methyluric acid, N-acetyl-L-leucine, and hexanoylglycine) were not yet, to our knowledge, related to TB. 9-methyluric acid is a methyl derivative of uric acid, and can result from the metabolization of different methylxanthines, such as caffeine, theophylline, and theobromine [37]. Its reduced serum levels were related to a prediabetes and metabolic syndrome [38]. N-acetyl-L-leucine is an acetylated derivative of leucine that is formed by a specific N-acetyltransferase or via the proteolytic degradation of N-acetylated proteins. It is used to treat neurodegenerative diseases [39]. Recently, it was described that N-acetyl-L-leucine levels are increased in hair and saliva of Type 2 diabetes patients [40,41]. Hexanoylglycine is an acyl glycine, a unusual minor metabolite of fatty acid resulting from glycine acyltransferase action, an important enzyme for xenobiotic detoxification, especially of benzoate and hydroxybenzoates [42].

In sum, in the present study, a signature of five metabolites (9-methyluric acid, trans-3-indoleacrylic acid, indole-3-lactic acid, hexanoylglycine, and N-acetyl-L-leucine) presenting maximum AUC, sensitivity, specificity, and accuracy was defined for the diagnosis of tuberculosis. Alternatively, a combination of metabolomic and proteomic biomarkers with equivalent performance was also proposed. This signature includes a protein, hemopexin, related with heme catabolism, and four of the above metabolites. Two of them (trans-3-indoleacrylic acid and indole-3-lactic acid) are involved in the metabolism of tryptophan, another (hexanoylglycine) in xenobiotic detoxification, and the fourth (N-acetyl-L-leucine) results from the proteolytic degradation of acetylated proteins. However, further investigation using larger cohorts, including other non-TB respiratory diseases, is needed. Additionally, the functional analysis of the differential levels of proteins and metabolites suggests the relevance of the complement system, cholesterol, iron, and tryptophan metabolism in the pathophysiology of TB infection.

## 4. Materials and Methods

### 4.1. Ethics Statement

This study was approved by the Heath Regional Administration of Lisbon and Tagus Valley Ethics Committee (ARSLVT, Proc.057/CES/INV/2014) and the National Commission for Data Protection (Portugal, N.5985/2014). Patients and healthy donors were recruited after signing informed consent.

### 4.2. Study Population

In this study, we used a subset of the cohort described in Conde et al. (2021). For the purpose of this study—both proteomic and metabolomic assays—a total of 67 individuals were included and subsequently divided into three experimental groups according to their clinical characteristics: 1. Healthy individuals with a negative IGRA test (control group); 2. Healthy, latently infected individuals who tested positive for the IGRA test (LTBI group); 3. Patients diagnosed with pulmonary and extrapulmonary tuberculosis (TB group). Inclusion criteria for the study were the following: age between 18 and 65 years old; no existence of other respiratory infection (in the case of participants with tuberculosis); HIV negative; diabetes negative; no chronic renal failure history; and individuals who have not been transplanted. Samples with signs of hemolysis or an undetermined result for IGRA test were excluded from this study. The demographic features of all subjects enrolled in this study are detailed in Table 2.

### 4.3. Study Design

For the proteomic assay, 63 participants were recruited and randomly divided into two sets, as depicted in Table 1: discovery set (n = 29), to identify possible biomarkers, and validation set (n = 34), to test the putative biomarkers. Of the 63 subjects enrolled in the proteomics assay, 45 were also studied in the metabolomic experiment, along with 4 new participants. Overall, the metabolomic set included 49 subjects. Each set was categorized into the same three experimental groups: control, LTBI, and TB. Figure 1 provides an overview of the study design and experimental workflow.

### 4.4. Sample Collection

Peripheral blood samples were collected at the Vendas Novas and Almada-Seixal Pneumonologic Diagnostic Centers (CDP-) following the procedure described in [13]. Briefly, for proteomic and metabolomic assays, around 7 mL of blood was collected in order to obtain 3 mL of serum. Whole blood samples were harvested using Clot Activator Tubes (Monovette Serum Gel Z—7.5 mL, S-monovette, Sarstedt^®^, Nümbrecht, Germany). IGRA test (QuantiFERON^®^-TB Gold IT, ©QIAGEN, Hilden, Germany) was used to confirm infection status in control and latent groups. These samples were transported at 4 °C to the National Institute of Health Doctor Ricardo Jorge (INSA). Samples displaying hemolysis or that had unidentified IGRA results were excluded from further analysis. Blood was allowed to clot for three hours at 4 °C after collection. The blood was then centrifuged at 1000× *g* at 4 °C for 30 min. Serum collected after centrifugation was passed through 0.2 μm filters (Sterile Acrodisc^®^, New York, NY, USA, syringe filters with Supor membrane, 32 mm) to remove bacteria. Finally, an antiprotease cocktail (Protease Inhibitor Cocktail, ©SIGMA, St. Louis, MO, USA) was added to the filtered serum. The samples were transported from INSA to ITQB in a liquid nitrogen container and stored at −80 °C.

### 4.5. Proteomic Assay

#### 4.5.1. Serum Immunodepletion and Protein Extraction

The six most abundant proteins in serum were depleted using the Multiple Affinity Removal Spin Cartridge Human 6 Kit (Agilent Technologies^®^, Santa Clara, CA, USA) following the manufacturer’s instructions. Protein was quantified with the bicinchoninic acid method (“QuantiPro™ BCA Kit”, Sigma-Aldrich^®^, St. Louis, MO, USA). The immunodepleted extract with 40 µg of protein was evaporated in the speedvac and the protein pellet was dissolved in 100 µL of 6 M urea 50 mM AB (ammonium bicarbonate) solution. Posteriorly, 1.4 µL of 700 mM DTT 50 mM AB was added, and the samples were incubated for 1 h at room temperature (RT). After this first incubation, 4.2 µL of 700 mM iodoacetamide in 50 mM AB solution was added to each sample, and a second incubation was performed in the dark for 30 min at RT. A third incubation for 15 min at RT followed the addition of 7.4 µL of 500 mM N-acetyl-L-cysteine in 50 mM AB. In order to dilute the urea, 486 µL of 50 mM BA solution was added to each sample. A 2 µL volume of Trypsin Gold, Mass Spectrometry Grade (Promega©, Madison, WI, USA) (1 µg/µL) was used to digest the proteins. All the samples were incubated overnight at 37 °C. To stop the digestion, 3 µL of formic acid (FA) was added. The digested samples were evaporated in the speedvac overnight. A 100 µL volume of 2% ACN in 1% FA solution was added to each sample. All the samples were further exposed to an ultrasonic bath for 20–25 min, with low amplitude. Peptide mixtures were desalted by C18 microcolumns (OMIX tips, Agilent Technologies, Santa Clara, CA, USA) following the manufacturer’s instruction, then vacuum-dried and maintained at −20 °C. The washed pellets were resuspended in 2% ACN and 0.1% FA, and 5 µL of a replicate of each experimental condition were used to create a pooled sample for protein identification. In order to remove insoluble material, the peptide mixtures were then centrifuged for 5 min at 14,000× *g*.

#### 4.5.2. SWATH-MS Analysis

Samples from the discovery and validation sets were analyzed on TripleTOF™ 5600 and 6600 Systems (ABSciex^®^, Toronto, ON, Canada), respectively, using information-dependent acquisition (IDA) of pooled samples for protein identification and SWATH-MS acquisition of each individual sample for protein quantification (as presented in [43]). Peptides were resolved by liquid chromatography (nanoLC Ultra 2D, Eksigent^®^, Redwood City, CA, USA) on a MicroLC column ChromXP™ C18CL (300 μm ID × 15 cm length, 3 μm particles, 120 Å pore size, Eksigent^®^) at 5 μL/min with a multistep gradient: 0–2 min linear gradient from 2 to 5%, 2–45 min linear gradient from 5% to 30%, and 45–46 min to 35% of acetonitrile in 0.1% FA and 5% DMSO. Peptides were eluted into the mass spectrometer using an electrospray ionization source (DuoSpray™ Source, ABSciex^®^) with 50 or 25 μm internal diameter (ID) hybrid PEELsil/stainless steel emitters (ABSciex^®^) in the case of the discovery and validation sets, respectively.

The pooled samples (one pool per group) were analyzed using the information-dependent acquisition (IDA) mode. The acquisition methods were adapted in accordance with the MS equipment used, in order to identify the largest number of proteins possible. Briefly, in the case of the discovery set, the mass spectrometer was set to scanning full spectra (*m/z* 350–1250) for 250 ms, followed by up to 100 MS/MS scans (*m/z* 100–1500 from a dynamic accumulation time—minimum 30 ms for precursor above the intensity threshold of 1000—in order to maintain a cycle time of 3.3 s). In the case of the verification set, samples were analyzed using two methods: a method with dynamic accumulation, as described before, and a conventional method. For the conventional method, the mass spectrometer was set to scan full spectra (*m/z* 350–1250) for 250 ms, followed by 30 MS/MS scans (*m/z* 100–1500) for 100 ms of accumulation time. In both methods, the candidate ions with a charge state between +2 and +5 and counts above a minimum threshold of 10 or 70 counts per second (for the dynamic or conventional method, respectively) were isolated for fragmentation, and one MS/MS spectra was collected before adding those ions to the exclusion list for 25 s (mass spectrometer operated by Analyst^®^ TF 1.7, ABSciex^®^). Rolling collision was used with a collision energy spread of 5.

For SWATH-MS-based experiments, both sets were analyzed with the same method. Briefly, the mass spectrometers were operated in looped product-ion modes [2] and the same chromatographic conditions used as in the IDA run described above. A set of 60 windows (Supplementary Table S5) of variable width (containing an *m/z* of 1 for the window overlap) was constructed, covering the precursor mass range of *m/z* 350–1250. A 250 ms survey scan (*m/z* 350–1250) was acquired at the beginning of each cycle for instrument calibration, and SWATH MS/MS spectra were collected from *m/z* 100–1500 for 50 ms, resulting in a cycle time of 3.25 s from the precursors, ranging from *m/z* 350 to 1250. The collision energy (CE) applied to each *m/z* window was determined considering the appropriate CE for a +2 ion centered upon this window, and the collision energy spread (CES) was also adapted to each *m/z* window.

Specific libraries of precursor masses and fragment ions were created, one for the discovery and another for the validation set, by combining all files from the IDA experiments, and used for subsequent SWATH processing of the respective set. Peptide identification and library generation were performed with ProteinPilot™ software (v5.1, ABSciex^®^) using the following parameters: (i) search against a database composed of Homo Sapiens from SwissProt (release in January 2017) and MBP-GFP; (ii) iodoacetamide alkylated cysteines as fixed modification; (iii) trypsin as digestion type. An independent false discovery rate (FDR) analysis, using the target-decoy approach provided by ProteinPilot™, was used to assess the quality of the identifications, and confident identifications were considered when identified proteins reached a 5% local FDR.

Data processing was performed using SWATH^TM^ processing plug-in for PeakView^TM^ (v2.0.01, ABSciex^®^). After retention time adjustment using the MBP-GFP peptides, up to 15 peptides, with up to 5 fragments each, were chosen per protein, and quantification was attempted for all proteins in the library file that were identified from ProteinPilot^TM^ searches. Peptides’ confidence threshold was determined based on an FDR analysis using the target-decoy approach, and those that met the 1% FDR threshold in at least three biological replicates were retained. The peak areas of the target fragment ions of those peptides were extracted across the experiments using an extracted-ion chromatogram (XIC) window of 4 or 6 min, for discovery and validation sets, respectively, with 100 ppm XIC width. The levels of the proteins were estimated by summing all the filtered transitions from all the filtered peptides for a given protein and normalizing to the total intensity obtained for samples.

The mass spectrometry proteomics data have been deposited into the ProteomeXchange Consortium via the PRIDE partner repository (Perez-Riverol et al. (2022)) with the dataset identifier px-submission #617008.

### 4.6. Metabolomic Study

#### 4.6.1. Metabolite Extraction

Serum samples (100 µL) were extracted with 300 µL of methanol overnight at 4 °C. On the next morning, the samples were centrifuged for 5 min at 16.000× *g* and 4 °C. The supernatant was transferred to a new tube, evaporated in the speedvac, and the pellet was stored at −20 °C until further analysis. Prior to LC-MS/MS analysis, the pellets were resuspended in 99.16 µL of H_2_O 0.1% FA. The Internal Standard (IS)—3-Nitro-L-tyrosine—was added to a final concentration of 42 µM. In addition, four pools of samples were used for metabolite identification.

#### 4.6.2. LC-MS/MS Analysis

LC-MS/MS data were acquired in a Dionex UltiMate 3000 UHPLC (ultra-high-performance liquid chromatography) system coupled to a QExactive Focus mass spectrometer using the Xcalibur v.4.0.27.19 software (Thermo Fisher Scientific, Waltham, MA, USA). For this analysis, the injection volume was 3 µL and the separation was performed using a Waters XBridge column C18 (2.1 × 150 mm, 3.5 µm particle size). A gradient of water with 0.1% formic acid (FA) (*v/v*) (A) and acetonitrile with 0.1% FA (*v/v*) (B) was applied as follows: 0–13 min, 1–99% B; 13–15 min, 99% B; 15–16 min, 99–1% B; 16–20 min, 1% B. The flow rate and the column temperature were kept at 0.4 mL/min and 30 °C, respectively. The eluate was infused into the MS through a heated electrospray (HESI) source. The parameters used were the following: sheath gas = 60 arbitrary units; aux. gas = 20 arbitrary units; sweep gas = 0 arbitrary units; spray voltage = 3 kV (positive polarity)/3.8 kV (negative polarity), capillary temp. = 320 °C; aux. gas heater temp. = 320 °C. Full-MS scan spectra were acquired in the *m/z* range 75–1125, at a resolution of 70,000 (full width at half maximum (FWHM) at *m/z* 200) and 1 *×* 10^6^ automatic gain control (AGC). Internal calibration was performed using lock mass at *m/z* 144.98215 and *m/z* 445.12003 (positive), and *m/z* 112.98550 (negative). To facilitate metabolite identification, a data-dependent MS/MS acquisition method was performed for each pool of samples, where the top 3 most intense ions were selected for higher-energy collisional dissociation (HCD). Stepped normalized collision energies (NCE) (20, 40, and 60) were applied, and MS/MS scan spectra were acquired at 17,500 resolution (FWHM at m/z 200), with 1 *×* 10^5^ AGC, maximum injection time of 100 ms, and dynamic exclusion of 6 s.

#### 4.6.3. Data Processing by Compound Discoverer 3.1™

Raw data files were independently processed by Compound Discoverer™ 3.1 (ThermoFisher Scientific) software for metabolomics data analysis. A blank sample was used for background subtraction and noise removal during the preprocessing step. A custom-designed workflow was set for spectra selection, alignment of retention times, compound detection and grouping, marking background compounds, and metabolite identification. The Detect Compound Node was set to detect XIC traces in the MS1 scans with a mass tolerance of 3 ppm and a minimal intensity threshold of 1,000,000. To group chromatographic peaks in compound grouping, the *m/z* width was 3 ppm and maximum retention time shift was set to 0.2 min. For background detection, the sample-to-blank ratio was set at 10. The preferred database used for metabolite identification was mzCloud—since the “Search mzCloud” node searches this database for matching fragmentation spectra (MS^2^)—followed by ChemSpider. For both databases, the mass tolerance that the software used to search for matching mass peaks was set at 3 ppm. In the case of the mzCloud search, the parameter “FT Fragment Mass Tolerance” was set to 5 ppm. This parameter determines the mass tolerance for high-resolution fragmentation scans carried out in the Orbitrap analyzer. The Human Metabolome Database (HMDB) was selected as the primary source for the ChemSpider search.

Several criteria were defined to accept the identification of a compound. “Full Match” in mzCloud was set as a mandatory condition; after functional evaluation, all compounds with exogenous origin were eliminated from further analysis: in positive and negative mode only compounds with “mzCloud Best Match” values higher than 9.5 and 9.8, respectively, were accepted.

Metabolomic data are available at the NIH Common Fund’s National Metabolomics Data Repository (NMDR) website, the Metabolomics Workbench, https://www.metabolomicsworkbench.org, where it was assigned Project ID ST003939.

### 4.7. Statistical and Bioinformatic Analysis

Statistical analysis was performed in Metaboanalyst (www.metaboanalyst.ca) [44]. Data were normalized by median, and Pareto was used for scaling. For the proteomic and metabolomic data, multivariate analysis was performed (principal component analysis (PCA), partial latent squares discriminant analysis (PLS-DA), and orthogonal PLS-DA (OPLS-DA)). Samples were normalized by median, and variables were scaled by Pareto. In the case of PLS-DA and OPLS-DA, a Q^2^ higher than 0.6 and a permutation test (2000) with *p* ≤ 0.05 were the thresholds for model acceptance. Variables with a VIP > 1.0 and 1.5 were considered important for group discrimination for proteomic and metabolomic data, respectively. In the case of proteomic data, univariate analysis was also performed. For the volcano analysis, we considered a fold change >2 and *p*-value adjusted by false discovery rate below 0.1.

Different receiver operating characteristic (ROC) curve analyses were performed between the control and TB groups. Firstly, they were used to ascertain the diagnostic capacity of each individual protein common to the two proteomic datasets: discovery and validation. The discovery set was used to construct the ROC, and the validation set was used as the prediction set. The proteins that presented an area under the curve AUC > 0.92 were selected in the analysis. All quantified metabolites were used for ROC analysis, and we selected five of those that showed an AUC > 0.9. Both the previously selected proteins and metabolites were used to perform an integrated ROC analysis, combining the proteomic and metabolomic data. The discriminant proteins were considered for the functional interactions network construction using STRINGdb (v11.0) (https://string-db.org). The determination of the power of the statistical analysis was performed with the R package MetSizeR: A Tool for Estimating Sample Sizes for Metabolomic Experiments (2021).

## Figures and Tables

**Table 1:** Proteins important for the discrimination between controls and TB patients in multi- and univariate analysis.

Uniprot Accession Number	Protein	VIP	Pathway
P01023	Alpha-2-macroglobulin	2.02	Complement coagulation cascade
P08603	Complement factor H	1.90	
P01024	Complement C3	1.70	
P02671	Fibrinogen alpha chain	1.58	
P05155	Plasma protease C1 inhibitor;	1.56	
P04003	C4b-binding protein alpha chain	1.51	
P01042	Kininogen-1	1.15	
P01031	Complement C5	1.14	
P02748	Complement component C9	1.03	
P00751	Complement factor B	1.00	
P02647	Apolipoprotein A-I	5.71	Cholesterol metabolism
P02652	Apolipoprotein A-II	1.63	
P27169	Serum paraoxonase/arylesterase 1	1.49	
P02749	Beta-2-glycoprotein 1	1.08	
P08519	Apolipoprotein(a)	1.10	
P02790	Hemopexin	5.79	Iron metabolism
P00450	Ceruloplasmin	3.08	
P02787	Serotransferrin	-	
P01011	Alpha-1-antichymotrypsin	3.75	
P04196	Histidine-rich glycoprotein	1.82	
P02763	Alpha-1-acid glycoprotein 1	1.71	
P02750	Leucine-rich alpha-2-glycoprotein	1.47	
P01019	Angiotensinogen	1.41	
P02766	Transthyretin	1.33	
P19827	Inter-alpha-trypsin inhibitor heavy chain H1	1.00	
P02774	Vitamin D-binding protein	1.00	

VIP values in grey indicate proteins increased in TB patients. Underlined protein names indicate proteins found in blood microparticles’ composition according to Gene Ontology.

**Table 2:** Demographic features of each of the cohorts enrolled in this study.

	Gender (*n*)			Age (Years)				BMI (kg/m^2^)				Smoker (*n*)		
	♂ (%)	♀ (%)	UNK (%)	♂ (± SD)	♀ (± SD)	Both (± SD)	UNK (n)	♂ (± SD)	♀ (± SD)	Both (± SD)	UNK (*n*)	Yes (%)	No (%)	UNK (*n*)
**Proteomic Cohort—Discovery Set (DS)**														
Control Group (*n* = 8)	4 (50%)	4 (50%)	-	42 (±12.3)	40 (±13.7)	41 (±12.1)	-	23 (±1.0)	23 (±6.5)	23 (±4.3)	-	1 (13%)	7 (87%)	-
LTBI Group (*n* = 7)	2 (29%)	5 (71%)	-	38 (±17.7)	52 (±13.0)	48 (±14.5)	-	21 (±1.6)	22 (±3.0)	22 (±2.6)	-	-	3 (43%)	4 (57%)
TB Group (*n* = 14)	5 (36%)	8 (57%)	1 (7%)	55 (±15.2)	46 (±15.3)	50 (±14.8)	1	24 (±2.5)	22 (±3.7)	23 (±3.3)	1	1 (7%)	9 (64%)	4 (29%)
**Proteomic Cohort—Validation Set (VS)**														
Control Group (*n* = 13)	5 (38%)	8 (62%)	-	44 (±14.6)	41 (±11.7)	42 (±12.3)	-	26 (±4.4)	25 (±4.5)	25 (±4.2)	1	1 (8%)	12 (92%)	-
LTBI Group (*n* = 3)	-	3 (100%)	-	-	45 (±6.1)	-	-	-	32 (±8.5)	-	-	-	1 (33%)	2 (67%)
TB Group (*n* = 18)	11 (61%)	5 (28%)	2 (11%)	41 (±12.6)	42 (±20.0)	40 (±14.4)	1	23 (±2.8)	27 (±2.4)	23 (±3.6)	-	5 (28%)	10 (56%)	3 (17%)
**Metabolomic Cohort**														
Control Group (*n* = 18)	8 (44%)	10 (56%)	-	44 (±13.5)	40 (±10.9)	42 (±11.9)	-	25 (±3.8)	24 (±4.6)	24 (±4.1)	1	2 (11%)	16 (89%)	-
LTBI Group (*n* = 6)	1 (17%)	5 (83%)	-	50 (NA)	52 (±10.0)	51 (±9.0)	-	20 (NA)	28 (±7.9)	27 (±7.8)	-	-	2 (33%)	4 (67%)
TB Group (*n* = 25)	13 (52%)	10 (40%)	2 (8%)	45 (±14.5)	43 (±15.2)	43 (±14.5)	1	23 (±2.7)	23 (±5.3)	23 (±4.0)	-	6 (24%)	13 (52%)	6 (24%)

Age and BMI are presented as the mean ± standard deviation. LTBI: latent tuberculosis infection; TB: tuberculosis; BMI: body mass index; UNK: unknown; *n*: number of subjects; NA: not applicable.

## Data Availability

The mass spectrometry proteomics data have been deposited into the ProteomeXchange Consortium via the PRIDE partner repository with the dataset identifier PXD037341. Metabolomic data are available at the NIH Common Fund’s National Metabolomics Data Repository (NMDR) website, the Metabolomics Workbench, https://www.metabolomicsworkbench.org, where it was assigned Project ID ST003939.

## Supplementary Materials

The following are available online at https://www.mdpi.com/article/10.3390/ijms232213733/s1.
