# Supplementary material for: Mass Spectrometry-Based Proteomic and Metabolomic Profiling of Serum Samples for Discovery and Validation of Tuberculosis Diagnostic Biomarker Signature
Source: Int J Mol Sci. 2022 Nov 8;23(22):13733. doi: 10.3390/ijms232213733 (PMC9694769; doi:10.3390/ijms232213733)
Supplement: Supplementary file 1 [file ijms-23-13733-s001.zip › Supplementary Figures.pptx]

## Slide 1
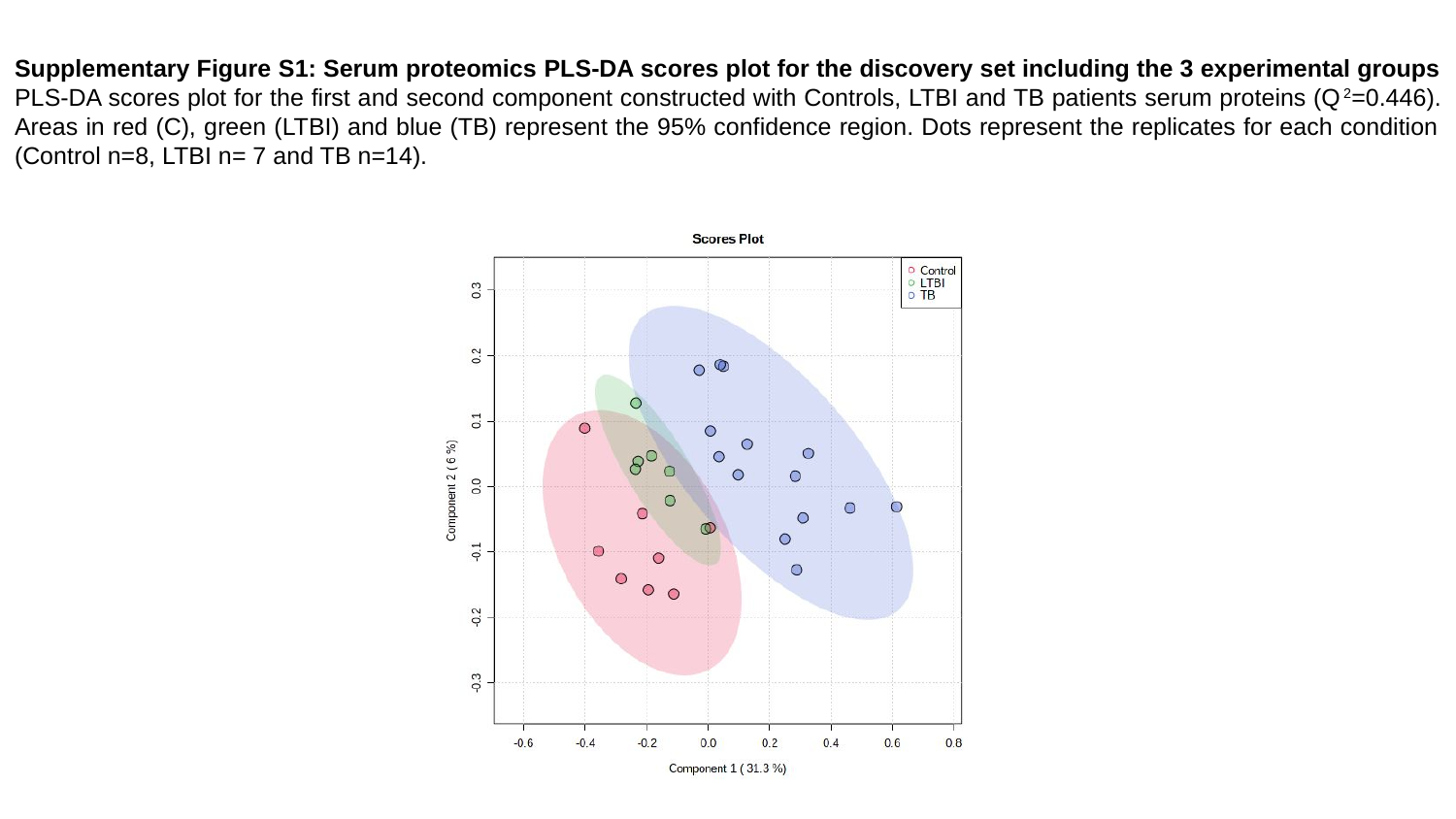

Supplementary Figure S1: Serum proteomics PLS-DA scores plot for the discovery set including the 3 experimental groups PLS-DA scores plot for the first and second component constructed with Controls, LTBI and TB patients serum proteins (Q2=0.446). Areas in red (C), green (LTBI) and blue (TB) represent the 95% confidence region. Dots represent the replicates for each condition (Control n=8, LTBI n= 7 and TB n=14).

## Slide 2
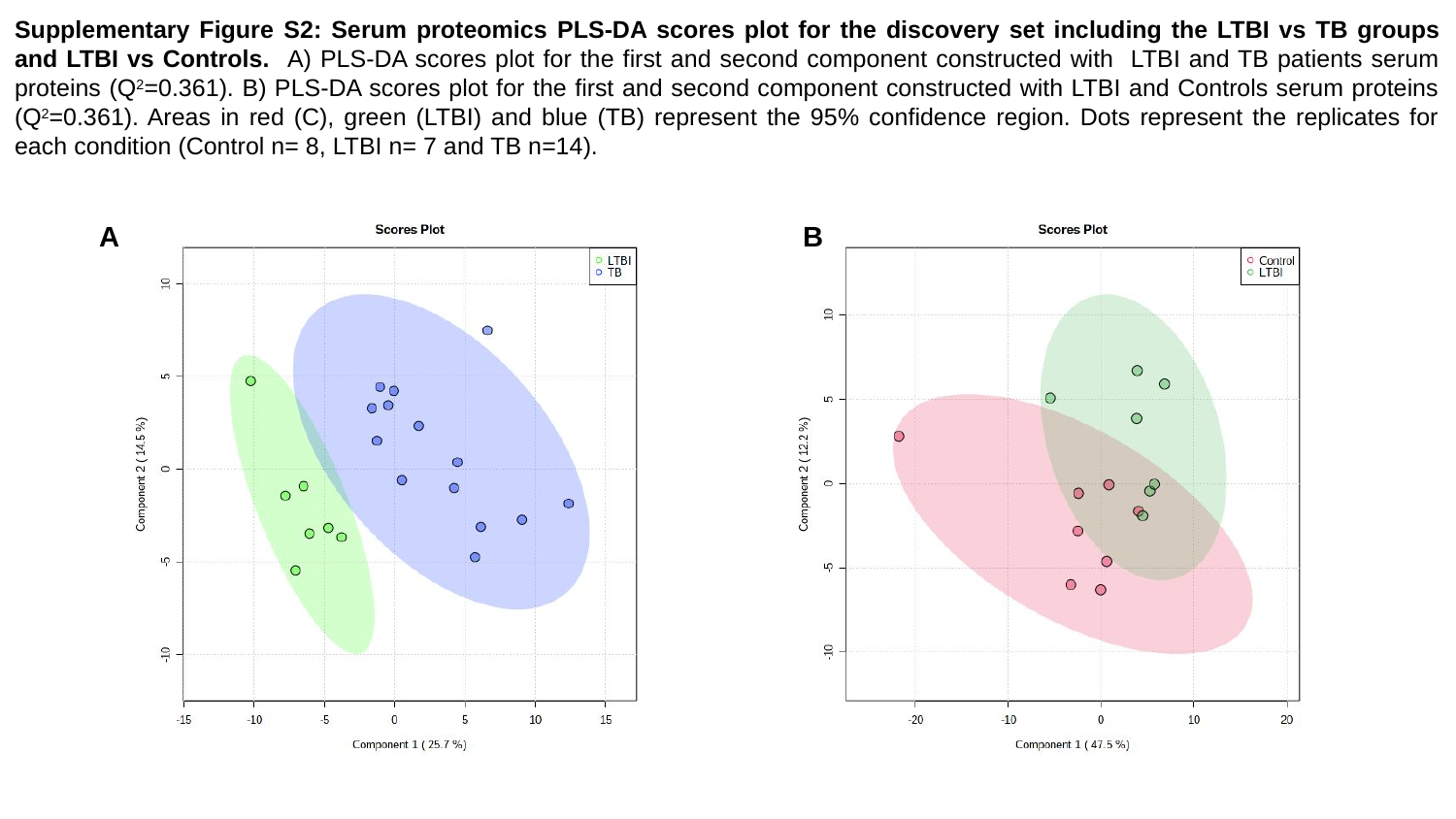

Supplementary Figure S2: Serum proteomics PLS-DA scores plot for the discovery set including the LTBI vs TB groups and LTBI vs Controls. A) PLS-DA scores plot for the first and second component constructed with LTBI and TB patients serum proteins (Q2=0.361). B) PLS-DA scores plot for the first and second component constructed with LTBI and Controls serum proteins (Q2=0.361). Areas in red (C), green (LTBI) and blue (TB) represent the 95% confidence region. Dots represent the replicates for each condition (Control n= 8, LTBI n= 7 and TB n=14).
A
B

## Slide 3
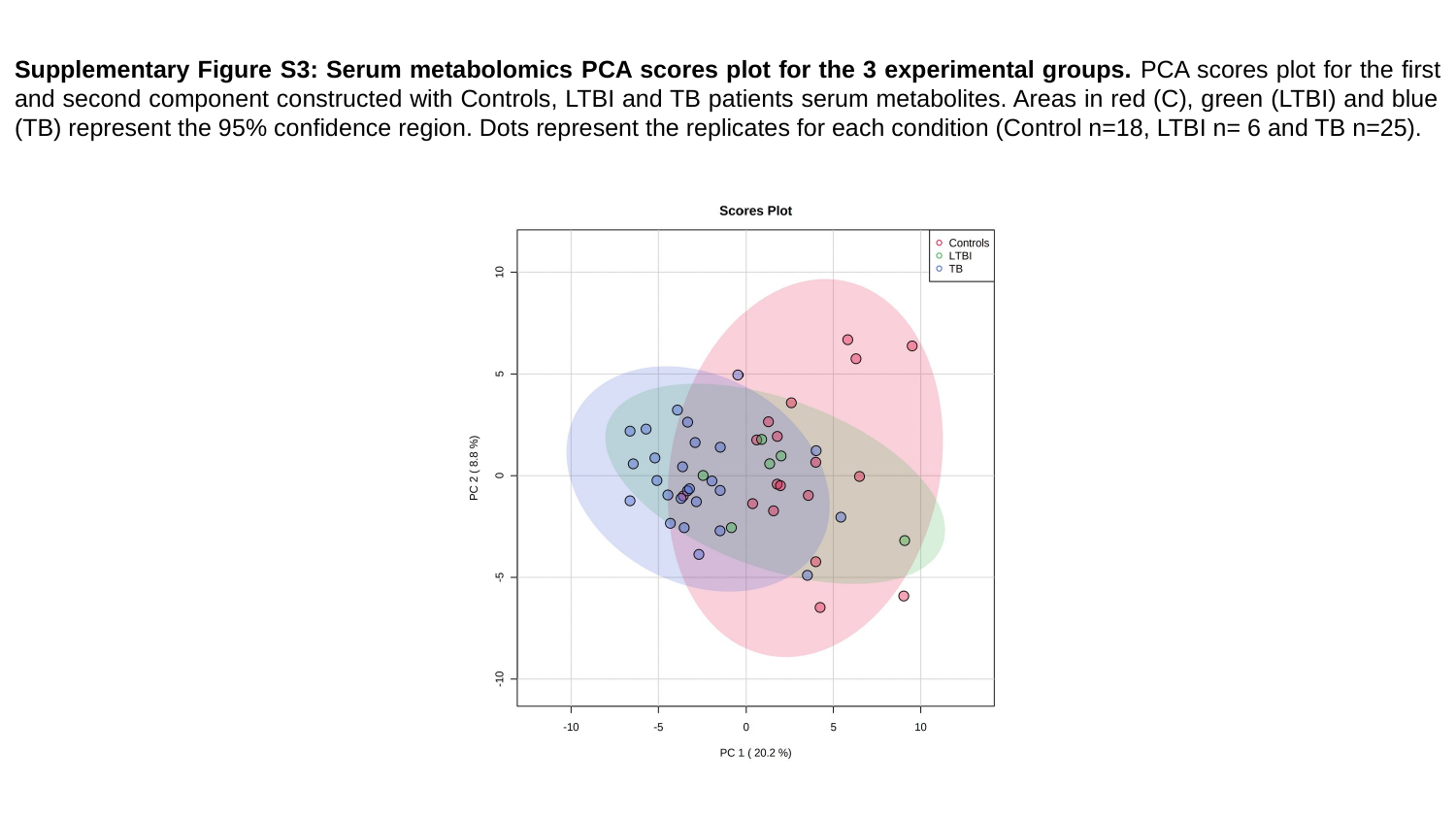

Supplementary Figure S3: Serum metabolomics PCA scores plot for the 3 experimental groups. PCA scores plot for the first and second component constructed with Controls, LTBI and TB patients serum metabolites. Areas in red (C), green (LTBI) and blue (TB) represent the 95% confidence region. Dots represent the replicates for each condition (Control n=18, LTBI n= 6 and TB n=25).
