# Supplementary material for: Mass Spectrometry-Based Proteomic and Metabolomic Profiling of Serum Samples for Discovery and Validation of Tuberculosis Diagnostic Biomarker Signature
Source: Int J Mol Sci. 2022 Nov 8;23(22):13733. doi: 10.3390/ijms232213733 (PMC9694769; doi:10.3390/ijms232213733)
Supplement: Supplementary file 1 [file ijms-23-13733-s001.zip › Table S5.pdf]

Table S5. SWATH-MS method. Set of *m/z* windows covering the precursor *m/z* range of 350-1250.

	m/z range	Width (Da)	CES
**Window 1**	349.5-358.6	9.1	5
**Window 2**	357.6-367.2	9.6	5
**Window 3**	366.2-376.6	10.4	5
**Window 4**	375.6-385.6	10	5
**Window 5**	384.6-395	10.4	5
**Window 6**	394-404.5	10.5	5
**Window 7**	403.5-413.5	10	5
**Window 8**	412.5-422.9	10.4	5
**Window 9**	421.9-433.3	11.4	5
**Window 10**	432.3-445	12.7	5
**Window 11**	444-457.6	13.6	5
**Window 12**	456.6-471.1	14.5	5
**Window 13**	470.1-485.5	15.4	5
**Window 14**	484.5-499	14.5	5
**Window 15**	498-512	14	5
**Window 16**	511-524.2	13.2	5
**Window 17**	523.2-535.9	12.7	5
**Window 18**	534.9-546.2	11.3	5
**Window 19**	545.2-557.1	11.9	5
**Window 20**	556.1-567.9	11.8	5
**Window 21**	566.9-577.8	10.9	5
**Window 22**	576.8-586.8	10	5
**Window 23**	585.8-594.9	9.1	5
**Window 24**	593.9-603	9.1	5
**Window 25**	602-611.1	9.1	5
**Window 26**	610.1-618.7	8.6	5
**Window 27**	617.7-626.4	8.7	5
**Window 28**	625.4-634.5	9.1	5
**Window 29**	633.5-642.6	9.1	5
**Window 30**	641.6-651.1	9.5	5
**Window 31**	650.1-661	10.9	5
**Window 32**	660-670.5	10.5	5
**Window 33**	669.5-679.9	10.4	5
**Window 34**	678.9-689.8	10.9	5
**Window 35**	688.8-699.3	10.5	5
**Window 36**	698.3-709.2	10.9	5
**Window 37**	708.2-718.6	10.4	5

**Window 38**	717.6-727.6	10	5
**Window 39**	726.6-738.4	11.8	5
**Window 40**	737.4-750.6	13.2	5
**Window 41**	749.6-761.8	12.2	5
**Window 42**	760.8-774	13.2	5
**Window 43**	773-786.1	13.1	5
**Window 44**	785.1-798.7	13.6	5
**Window 45**	797.7-810.9	13.2	5
**Window 46**	809.9-823	13.1	8
**Window 47**	822-836.5	14.5	8
**Window 48**	835.5-850.9	15.4	8
**Window 49**	849.9-867.1	17.2	8
**Window 50**	866.1-886.5	20.4	8
**Window 51**	885.5-905.8	20.3	8
**Window 52**	904.8-924.7	19.9	8
**Window 53**	923.7-944.5	20.8	8
**Window 54**	943.5-966.6	23.1	8
**Window 55**	965.6-998.1	32.5	8
**Window 56**	997.1-1033.6	36.5	10
**Window 57**	1032.6-1066.5	33.9	10
**Window 58**	1065.5-1103.4	37.9	10
**Window 59**	1102.4-1165	62.6	10
**Window 60**	1164-1249.6	85.6	10
